# FRAILTY TRAJECTORY AMONG VETERANS WITH HEART FAILURE AGED 50+: 20-YEAR LONGITUDINAL STUDY

**DOI:** 10.1093/geroni/igad104.2768

**Published:** 2023-12-21

**Authors:** Javad Razjouyan, Aanand Naik

**Affiliations:** Baylor College of Medicine, Houston, Texas, United States; University of Texas Health Science Center at Houston, Houston, Texas, United States

## Abstract

A few studies used large longitudinal cohort of heart failure (HF) patients to report the shape of frailty trajectory (FT) and its importance in predicting death. We assessed the additive predictive power of FT to the cross-sectional frailty index (FI). A retrospective cohort of veterans aged 50+ by 10/2022 were constructed. We included patients admitted with HF for the first time as the principal diagnosis; had two primary care physician visits in for each year in past three years; admitted after 2001 and had reported ejection fraction at the time of admission. FI constructed based on the previously validated FI for veterans. The three FIs for each year before admission were used to fit a linear line model. The slope and the intercept were used to characterize the FT. We used logistic regression to compare the predictivity of FI versus FT by reporting area under the curve (AUC) for 30-day mortality and all-cause mortality. 111,426 included with median follow-up of 3.0 (IQR,1.4,5.3) years. 82% died within the first 5 years (age,72±10;BMI,31±8; EF≤40%,48%). On average 1.8 per year deficits accumulated annually and 78% of patients had FI≥0.2 at the time of admission versus 60% in a year prior to admission. FT improve the AUC of 30-day and all-cause mortality by 6% compared to FI. FT is a better predictor of mortality than cross-sectional FI for HF population. FT provided additional information beyond the cross-sectional FI by enhancing mortality prediction, identifying individuals who are at high risk of mortality with low FI.

